# Rule-Based Design of Plant Expression Vectors Using GenoCAD

**DOI:** 10.1371/journal.pone.0132502

**Published:** 2015-07-06

**Authors:** Anna Coll, Mandy L. Wilson, Kristina Gruden, Jean Peccoud

**Affiliations:** 1 Department of Biotechnology and Systems Biology, National Institute of Biology, Ljubljana, Slovenia; 2 Virginia Bioinformatics Institute, Virginia Tech, Blacksburg, Virginia, United States of America; McGill University, CANADA

## Abstract

Plant synthetic biology requires software tools to assist on the design of complex multi-genic expression plasmids. Here a vector design strategy to express genes in plants is formalized and implemented as a grammar in GenoCAD, a Computer-Aided Design software for synthetic biology. It includes a library of plant biological parts organized in structural categories and a set of rules describing how to assemble these parts into large constructs. Rules developed here are organized and divided into three main subsections according to the aim of the final construct: protein localization studies, promoter analysis and protein-protein interaction experiments. The GenoCAD plant grammar guides the user through the design while allowing users to customize vectors according to their needs. Therefore the plant grammar implemented in GenoCAD will help plant biologists take advantage of methods from synthetic biology to design expression vectors supporting their research projects.

## Introduction

Synthetic biology aims at bioengineering organisms that perform beneficial functions, generally by means of a rational design approach [1]. Plants have largely been unexploited for synthetic biology, but they offer great potential [2]. To fully benefit from synthetic biology, significant efforts have been dedicated to the development of robust, less-demanding, and more reliable methods to assemble increasingly complex designs (see review [3]). Beyond the assembly of constructs, the design of complex multigene vectors is a big challenge. Editing large DNA sequences increases the risk of introducing errors. Furthermore, identifying suitable biological parts is becoming more difficult as the number of parts for synthetic biology increases. Therefore, there is a need for software tools that guide plant synthetic biologists through the design of application-specific expression vectors. GenoCAD (www.genocad.com) is a Computer-Aided Design (CAD) software for synthetic biology which allows the user to quickly design protein expression vectors, artificial gene networks and other genetic constructs based on the notion of genetic parts [4]. GenoCAD includes a system to manage annotated and user-defined genetic parts. Moreover, it also guides the users through the design by means of a set of predefined rules that describe the design strategy for a specific type of application and which can be expressed in a context-free grammar [5]. By default, GenoCAD includes a simple grammar used for demonstration purposes. However, the grammar editor embedded in GenoCAD enables users to develop brand new grammars, and therefore provides biologists with a tool to formalize custom design strategies. Several grammars have already been added by users i.e. (i) a grammar to design a family of vaccine vectors derived from vesicular stomatitis virus (VSV) [6], (ii) a grammar to design *Chlamydomonas reinhardtii* expression vectors [7] and (iii) a grammar to design synthetic transcription factors in eukaryotes [8].

Here, we present a vector design strategy to express genes in plants. Specifically, we developed a grammar to design constructs for three categories of experiments: promoter analysis, protein localization and protein-protein interaction (PPI) studies, which are perhaps the most demanding in plant biology. The design rules of these vectors are sufficiently different from the design of protein expression in *E*.*coli* to justify the development of a new grammar following the grammar-design workflow previously described by Wilson et al [7]. Constructs for promoter analysis, localization, and PPI studies are frequently used in the plant biology community. As can be seen in the literature they are the methods of choice to functionally characterize plant proteins. For instance, among other examples, these methodologies were used to elucidate the role of MAPKKKε in plant immune response against *Phytophthora infestans* [9], to study the function of ERF3 in potato response to various biotic and abiotic stresses [10] or to understand the role of *Arabidopsis* WRKY8 in mediating salt stress tolerance [11]. Many commercially available plasmids can be used for these applications, but they do not always meet all the requirements of specific projects. This usually forces the user to adapt the vector, and edit the sequence using a cut-and–paste approach, which has a high chance of introducing errors. Here we have captured the expertise gained by our group over the years to produce set of design rules and a parts library, which will guide non-expert users through the design process. Advanced users can use the GenoCAD grammar editor to customize this grammar by adding rules and parts to extend the scope of the grammar. Alternatively, they could also simplify the grammar by eliminating specific rules that do not apply to their projects, or even delete an entire branch of the grammar to streamline the design process.

## Methods

### Category definition

First, we identified different categories of genetic parts used to design plant expression vectors (S1 File). Our plant grammar includes several general categories found in many other grammars e.g. gene (GEN), epitope tag (ETG), fluorescent tag (FTG), linkers (LNK), terminator (TER) or promoter (PRO) (S1 Table). The category PRO refers to the most commonly used promoters for expression in plants. These are constitutive and strong promoters usually of viral origin. In this plant grammar we distinguished them from native promoters (NPRO), which includes promoters naturally found in plants that regulate the expression of plant genes. Like for the *C*. *reinhardtii* chloroplast grammar [7], start (ATG) and stop (STP) codon are considered as categories in order to facilitate the design of fusion proteins. Since 5’UTR and 3’UTR are not well characterized in plants, here they are included in the promoter and terminator category respectively.

In addition to terminal categories corresponding to groups of genetic parts, a grammar generally includes rewritable categories corresponding to higher levels of organization. For instance, we defined the categories ‘one/two promoter’ (PRO12) and ‘one/two terminator’ (TER12) to allow the option of using a single or double promoter and terminator. We also defined categories specific to each application (S1 Table). For instance, GFTG is a rewritable category consisting of a gene fused to a FTG. It is a specific category for protein localization studies. These categories will be explained in detail in Results.

The grammar also includes categories used as delimiters. Parentheses are used to delimit the sequence of a plasmid. They can be used to indicate that a design corresponds to a complete plasmid. They can also be used in situations where an experiment calls for a co-design of two plasmids working together.

The brackets [] are used to indicate the orientation of a sequence. Elements between brackets are coded on the negative strand. DNA sequences of elements between brackets are reverse-complemented when exported as a text file.

### Rules

Rules for the plant grammar (S1 File) are shown in S2 Table. *S* is the start symbol, and it can be rewritten into an expression plasmid for localization studies (*loc* route), for promoter analysis (*pro* route) or into plasmids for PPI studies (*ppi* route). There are several common rules for *ppi* and *loc* routes to rewrite the GEN, PRO and TER categories. There are 5 rules to rewrite the GEN category. Rule *2gen* is used to fuse two proteins. Rules *tgen* and *gent* allow expressing a protein fused to an ETG on its N or C terminus. Epitope tags are widely used for detection or purification purposes. The rules *lgen* and *genl* are used to include a LNK between fusion proteins. Linkers are routinely used when expressing recombinant fusion proteins in order to improve expression, folding and stability of the proteins [12]. Three rules can be used to rewrite the PRO12 category; rule *pro1* is used to add a single promoter, rule *pro2* allows the user to introduce an additional promoter, and rule *npro* introduces a native promoter. Similarly, the TER12 category can be rewritten into either a single or double terminator. This rule is also applicable to the route for promoter analysis. Moreover, *pro* and *loc* routes share rules to rewrite the FTG category. The fluorescent tag can be rewritten in both cases as a protein with epitope tags and/or linker domains at its N and/or C terminus.

Specific rules for each route are described in the Results section.

### Parts Library

The library was built by importing sequences into each category (S1 File). The sources of part sequences are available in the parts description field. Under GEN and NPRO categories we imported sequences from *S*. *tuberosum* group Phureja DM1-3 [13] as an example since these are the sequences experimentally tested in our laboratory.

The use of FTGs in plant biology is especially challenging because of the autofluorescence displayed by several components of plant tissues (chlorophyll, lignified secondary cell walls, and vacuolar contents) overlap with the emission wavelength of green fluorescent protein (GFP) [14]. The FTGs currently added in the GenoCAD parts library have all been previously tested in *Nicotiana benthamiana* and *Solanum tuberosum* leaves, and they include enhanced GFP (eGFP), yellow fluorescent protein (eYFP), cyan fluorescent protein (eCFP) and mCherry.

Several combinations of fluorescent protein fragments have been functionally tested and recommended for bimolecular fluorescence complementation (BiFC) analysis [15]. All of them present advantages and drawbacks in terms of complementation efficiency and fluorescence intensity. Fragments of YFP truncated at residue 155 [16] were incorporated in the parts library because, according to Kerppola et al. [17] and our own experience, though having weak fluorescent intensity, this combination of FTG fragments exhibits low spontaneous association, and, therefore, less possibility of false positive results than others.

Vector backbones to clone the assembled parts currently available in the library include four different pCAMBIA minimal selection vectors (http://www.cambia.org). These vectors are T-DNA binary vectors (see review [18]), and are therefore compatible with Agrobacteria-mediated plant transformation. They contain minimal heterologous sequences for plant transformation and selection of transformants (in bacteria and plant); they allow for the insertion of desired genes for transformation into plants, but require all regulatory sequences for plant expression of newly cloned genes. All vectors were opened at the multiple cloning site (MCS) with *SalI* and *BamHI* resitriction enzymes.

## Results

Plant transformation for functional analysis experiments has become a routine tool in plant research. Nowadays, commercial plant expression plasmids for different applications are available. However, this is a rigid option, and to change any element of the backbone plasmid is a time-consuming task. Moreover, most of them are based on classical or Gateway cloning systems. There is a need for simple and versatile design strategies to allow high throughput approaches in synthetic biology studies. Therefore, we developed a GenoCAD grammar to design constructs for *in planta* functional analysis studies (S1 File). The plant grammar is available in Figshare (doi.org/10.6084/m9.figshare.1428589).

### Design of expression plasmids for protein localization studies

Studying the protein subcellular localization in living plant cells is a useful tool for characterization of unknown proteins. Fusion of fluorescent tags to proteins of interest has become the method of choice for this purpose [19]. The availability of FTG markers has increased noticeably over the last two decades. The large selection of FTG markers available in plant molecular biology together with the development of sophisticated image acquisition and analysis software contributed to the rapidly and increased adoption of this technology by plant molecular biologists [14]. Therefore, we implemented a grammar to design constructs for protein localization studies based on fused FTG.

**Fig 1:**
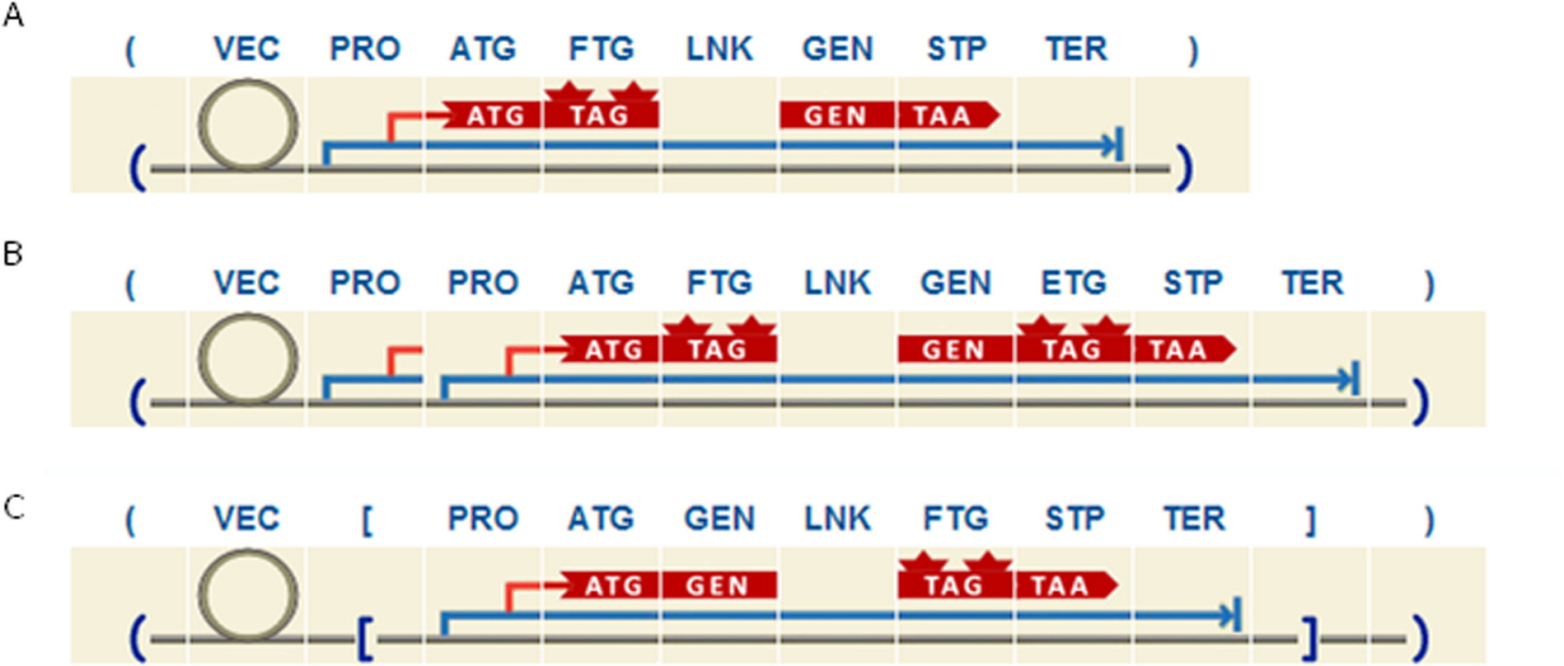
Example of three different designs for localization studies purposes as developed with the plant grammar. A. Scheme of the most basic structure we can design, where the expression cassette includes the GEN fused to a FTG by means of a LNK domain on the N terminal. B. Sample design includes an expression cassette with 2 PRO and a GEN fused to a FTG on the N terminal and to an ETG on the C terminal. C. Same as B but with the expression cassette in reverse orientation.

Route *loc* guides the user through the design of plant expression vectors for localization studies (S2 Table). By means of *lcas* rule, localization category (LOC) is rewritten into a complete plasmid that includes an expression cassette (CAS) and vector backbone (VEC). The next rule is *prct*, which breaks the cassette down into PRO12, open reading frame (CDS) and TER12. The orientation of the expression cassette can be reversed by using *rcas* rule. Rules *npro*, pro1, *pro2*, *ter1* and *ter2* allow the user to design an expression cassette with a native promoter, single or double promoter, and single or double terminator. Afterwards, rule *gnftg* break the CDS down into an ATG, GFTG, and STOP codon. Therefore, this rule constrains the user to design an expression cassette with a gene fused at least with one fluorescent protein, which is the minimal requirement for plasmids with localization application purposes. Two rules (*nftg* and *cftg*) incorporate flexibility into the design, allowing users to add the fluorescent protein on the 5’ or 3’-end of the gene. Moreover, epitope tags and/or linker domains can also be added at both sides of the FTG (rules *tftg*, *ftgt*, *lftg* and *ftgl*). Lastly, as it was described, GEN category can be rewritten in order to add other open reading frames, epitope tags and/or linkers at the 5’ and/or 3’-end of the gene. To demonstrate the flexibility of this approach, 3 examples of different designs with different degrees of complexity, all of them applicable for protein localization studies, are shown in Fig 1.

### Design of promoter analysis studies

Identification and characterization of plant promoters is crucial to understanding the function of the genes under their control. Characterizing the promoters themselves is also important because they are valuable tools in plant genetic engineering (see [20] as an example). Therefore, the promoter-reporter analysis is an indispensable approach for molecular plant biologists. In our GenoCAD plant grammar, we implemented a set of rules to direct the user though the design of plant expression vectors suitable for promoter studies (route *pro*; S2 Table).

**Fig 2:**
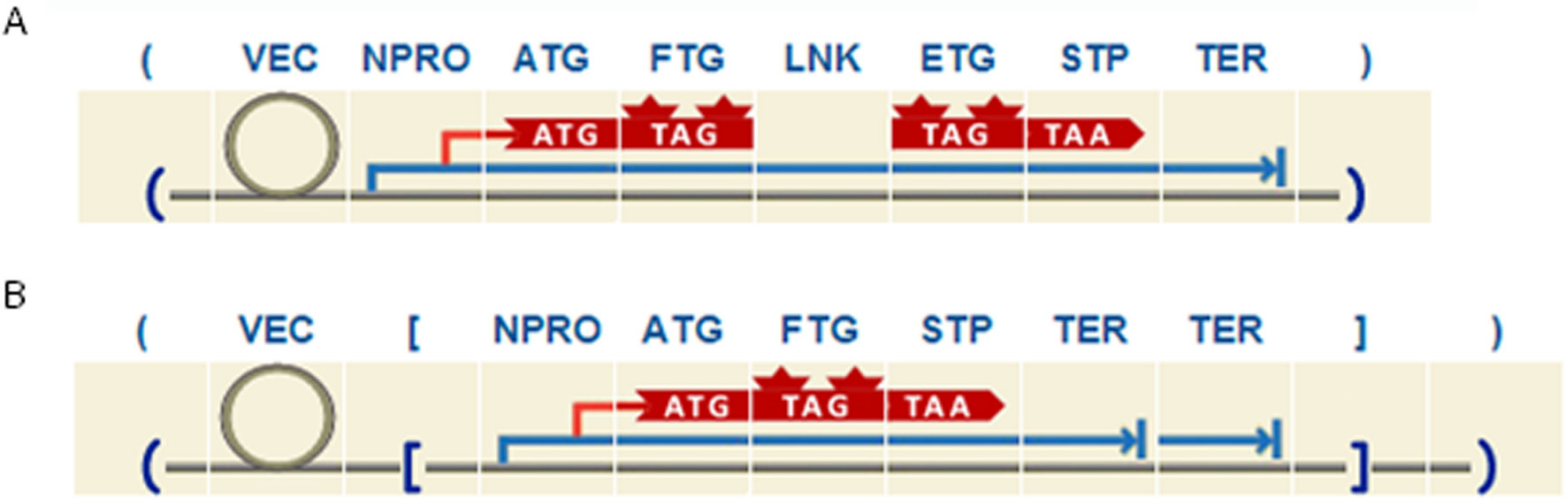
Example of two different designs for promoter analysis studies. A. The expression cassette includes a FTG under the control of a NPRO and fused with an ETG by means of a LNK. B. The expression cassette has reverse orientation and double TER.

The main requirement of promoter-reporter systems is that the sequence of the promoter of interest (native promoter) has to be fused to a reporter protein. To that end, the rule *npct* breaks down the expression cassette into a NPRO followed by a FTG and TER12. The rule *rnpct* allows the user to clone the expression cassette in reverse orientation. Moreover, using the same rules mentioned above a single or double terminator can be added to the cassette, and epitope tags and/or linker domains can be included at both sides of the FTG. As an example, Fig 2 shows 2 different designs suitable for promoter analysis studies.

Since plant promoter information is still scarce, so far the compilation of native promoter sequences in the plant GenoCAD library is based on identification and characterization of several *Solanum tuberosum* promoters performed in our research group (e.g. StMKK6 promoter [21]). However, the idea is that each user expands the library with new entries of experimentally characterized promoters of interest [22].

### Design expression plasmids for protein-protein interaction studies

PPI plays a crucial role in regulating biological systems, and provides valuable information about protein functions. In recent years, large-scale PPI studies have been applied to plants and allowed us to better understand the complex networks through which plant proteins exercise their functions (see reviews [23–25]). As an example, *Arabidopsis* interactome map [26] is the most complete interactome network published nowadays. Independently of how carefully large PPI experiments are performed, at least a representative subset of the data should be validated using an independent assay [27]. On the other hand, *in planta* functional validations of specific interactions are still necessary to understand the function of specific proteins of interest. Such experiments usually require the application of a combination of methods to a particular biological system. Over the last 10–15 years, many methods to characterize interactions in more realistic *in planta* settings have been developed (see review [24]). BiFC and targeted co-immunoprecipitation (CoIP) are the preferred choice for most of the researchers [28–30].

**Fig 3:**
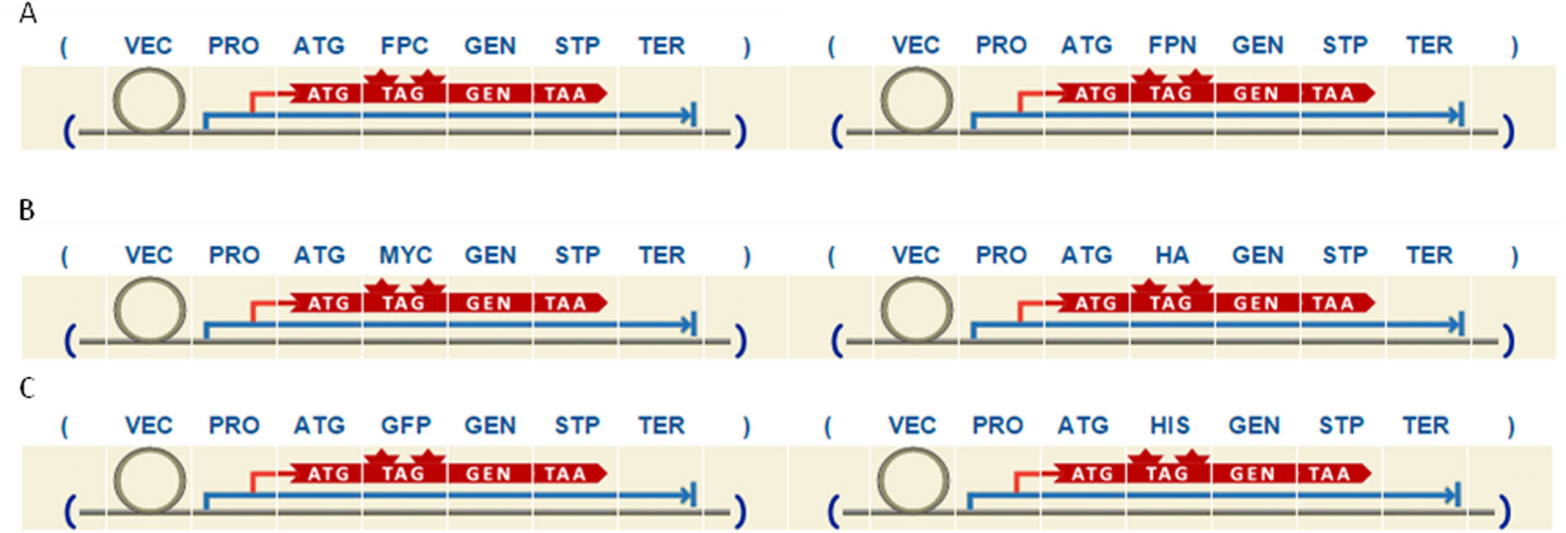
Example of three different designs obtained following *ppi* route. A) *bifc* route. B) *coip* route using MYC and HA as epitope tags. C) *coip* route using GFP and HIS as epitope tags.

In BiFC approaches, two potential interacting proteins are fused to two fragments of a fluorescent protein, and identification of interactions is based on the reconstitution of the split fluorescent tag [31]. In the CoIP assay, the protein complex is precipitated with an immobilized antibody against one of the proteins studied, and the interacting partner is further confirmed by Western Blot. Usually antibodies against tagged fusion proteins are used [32]. According to the principle of these methodologies, the most important specificity of both studies is that 2 vectors have to be designed in parallel to allow proper experimental design. A split fluorescent protein is required in vectors for BiFC studies, and 2 different epitope tags in the case of the CoIP approach. To satisfy these requirements, the *ppi* route implemented in our grammar gives the users two options i.e. *bifc* and *coip* route (S2 Table). In the first case (Fig 3A), the rule defines that the design involves 2 plasmids, the first one containing the C-terminal of a half-fluorescent protein fused to the target protein (GFPC), and the second one containing the N-terminal of the same fluorescent protein (GFPN). Epitope tags and/or linker domains can also be added at both sides of the fluorescent protein (rules *tfpc*, *fpct*, *lfpc*, *fpcl*, *tfpn*, *fpnt*, *lfpn*, *fpnl*). On the other hand, the *coip* rule defines that the design involves 2 plasmids containing two different epitope tags fused to the potential interacting proteins. According to the most commonly used tags in plant molecular biology, two-pair combinations of epitope tags are included in the grammar i.e. c-myc (MYC)/Human influenza hemagglutinin (HA) and GFP/poly-histidine (HIS) tag. Specifically, rule *myha* defines that the first plasmid contains a cassette, which includes a gene fused to MYC epitope tag (GMY), and the second plasmid contains the gene fused to the HA epitope (GHA) (Fig 3B). In the same way, rule *gfhi* forces the user to design the first plasmid with GFP fused to the gene (GGF), and the second plasmid with a gene fused to HIS tag (GHI) (Fig 3C). As in the case of localization studies, GEN category allow the user to add epitope tags and linkers at both sides of the protein under study, giving again high flexibility to the design.

## Discussion

We implemented a plant grammar in GenoCAD, which guides the user through the design of plant expression vectors for functional studies experiments. The PPI branch of the grammar includes rules that express the requirement that genetic parts located on different plasmids interact with one another. For instance, rules describing the use of split fluorescent proteins to visualize the interactions between two proteins require two plasmids to use sequences complementing each other. This type of systems-level analysis relying on trans-interactions between genetic parts had been proposed before [33] but this is the first time that this design constraint is captured in a grammar.

An important aspect of the grammar development effort was the development of a consistent set of icons suitable to properly represent the structure of the genetic constructs. We first attempted to use the icons of the Synthetic Biology Open Language (SBOL) in order to adhere to this emerging community standard [34]. SBOLv, the visualization component of the SBOL effort, currently includes 21 glyphs, 11 of which corresponding to functional elements as 10 icons are used to represent structural elements like restriction sites. Since our grammar includes 44 different categories, we could not find a satisfactory way to map our categories to the set of SBOLv icons. At some point, it seemed preferable to develop a set of custom icons. The icons have been designed to be consistent with one another so as to show the DNA, RNA, and proteins associated with different DNA sequences. The development of this grammar illustrates the conflicting requirements to develop a controlled vocabulary customized for a particular domain of application with the need to promote standards accepted by a large scientific community [35].

Even though it is possible to include in a grammar rules associated with specific assembly standards [22], rapid progress in DNA synthesis [36] makes it possible to assume users of this grammar would be able to obtain any sequence designed with the grammar by means of sequence-independent processes using the services of a DNA synthesis company or assembling these sequences in house [37].

The grammar we developed is based on our current experience in plant expression vectors. Rules were established according to the specifications required for specific plant functional analysis-oriented applications in order to design functional vectors that can be used for a wide range of plant species as long as they are transformable by *Agrobacteria tumefaciens*. We covered three types of applications i.e. protein localization, PPI and promoter analysis, which are indispensable and commonly used experiments among the plant biology community to discover and characterize gene functions.

The next step is now validation of constructs presented in the grammar since the complete final plasmids have not yet been experimentally verified for functionality. However, in our laboratory, all library parts have been tested in *Nicotiana benthamiana* using commercially available plasmids. As an example, for localization studies, the plant binary Gateway vector pH7YWG2 which allows the expression of translational fusions with YFP [38] was modified and validated to express *Solanum tuberosum* mitogen-activated protein kinase (StWiPK) fused to mCherry.

The use of GenoCAD will certainly reduce time and cost of our experiments. For instance, the aforementioned simplest construct for localization studies (i.e. protein fused to a fluorescent tag) involves choosing between 3 different fluorescent tags at either N or C-terminal position bringing in 6 different plasmids. With the use of GenoCAD there is no need of a large amount of different commercial plasmids which meets all different experiment requirements. One single plasmid can be used to clone the different variant cassettes that better fulfill our needs with no plasmid modifications required and therefore fewer chances of errors during sequence manipulation.

The grammar is flexible enough to meet users’ specific needs. It can generate constructs that may have questionable features, like a gene fused to several peptide linker sequences. We could have defined stricter constraints, such as imposing a linker sequence between protein domains. However, it has been recently reported that linker sequences are not critical factors for the success of BiFC experiments [29], therefore we decided to favor flexibility and let the user decide between different design options. If the need for additional features is identified by the user, the grammar can be revised and improved to encompass developments in the field.

In short, we presented a grammar implemented in GenoCAD to guide users through the design of plant expression vectors. The grammar is divided into different modules according to the aim/application of the final design. As an example, we developed rules for 3 of the main applications in plant functional studies: protein localization, promoter analysis, and PPI studies. We also curated a library of basic parts associated with the plant grammar, where each part is categorized into functional groups. The GenoCAD grammar editor makes it relatively easy to modify the grammar. A natural extension of our plant grammar would be to add rules for designing multigenic constructs, and also for other applications potentially interesting for plant biologists, e.g. silencing (VIGS, artificial microRNAs).

Taken altogether, this GenoCAD library will facilitate the design of large plant constructs that can be obtained using sequence-independent methods. It will contribute to transitioning methods from synthetic biology into mainstream plant science.

## Supporting Information

S1 File Plant Grammar. (ZIP)

S1 Table Categories of genetic parts used in the plant grammar. (XLS)

S2 Table The rules for plant grammar. (XLS)
