# Correction: Rule-Based Design of Plant Expression Vectors Using GenoCAD

**DOI:** 10.1371/journal.pone.0136004

**Published:** 2015-08-13

**Authors:** Anna Coll, Mandy L. Wilson, Kristina Gruden, Jean Peccoud

The following information is missing from the Competing interests section: Jean Peccoud holds a financial interest in GenoCAD and serves as the company’s CEO. This affiliation does not alter the authors' adherence to all the PLOS ONE policies on sharing data and materials.

GenoCAD did not provide any funding for this study and was not involved in the design of the research, the collection of data, or the preparation of the manuscript.
